# Comparative SILAC Proteomic Analysis of *Trypanosoma brucei* Bloodstream and Procyclic Lifecycle Stages

**DOI:** 10.1371/journal.pone.0036619

**Published:** 2012-05-04

**Authors:** Michael D. Urbaniak, M. Lucia S Guther, Michael A. J. Ferguson

**Affiliations:** Division of Biological Chemistry and Drug Discovery, College of Life Sciences, University of Dundee, Dundee, United Kingdom; University of Texas-Houston Medical School, United States of America

## Abstract

The protozoan parasite *Trypanosoma brucei* has a complex digenetic lifecycle between a mammalian host and an insect vector, and adaption of its proteome between lifecycle stages is essential to its survival and virulence. We have optimized a procedure for growing *Trypanosoma brucei* procyclic form cells in conditions suitable for stable isotope labeling by amino acids in culture (SILAC) and report a comparative proteomic analysis of cultured procyclic form and bloodstream form *T. brucei* cells. In total we were able to identify 3959 proteins and quantify SILAC ratios for 3553 proteins with a false discovery rate of 0.01. A large number of proteins (10.6%) are differentially regulated by more the 5-fold between lifecycle stages, including those involved in the parasite surface coat, and in mitochondrial and glycosomal energy metabolism. Our proteomic data is broadly in agreement with transcriptomic studies, but with significantly larger fold changes observed at the protein level than at the mRNA level.

## Introduction

*Trypanosoma brucei* is a protozoan parasite transmitted by the bite of the tsetse fly, and is the etiological agent of African sleeping sickness. The disease is invariably fatal if untreated and is estimated to be responsible for ∼10,000 deaths per annum in sub-Saharan Africa [1]. Current treatments are expensive, toxic and difficult to administer leaving an urgent unmet need for new therapeutic agents [2].

*T. brucei* has a complex digenetic lifecycle between an insect vector and mammalian host, and the ability to respond to its environment through adaption of its proteome is essential to its survival and virulence. The clinically relevant bloodstream form lives in the bloodstream and lymph of the host in the first stage of the disease, before crossing the blood-brain barrier in the second stage of the disease leading to coma and death. The pleomorphic bloodstream form exists as both a replicative long-slender morphology and a division arrested stumpy form which is pre-adapted for transmission into the tsetse fly. Upon ingestion by the tsetse fly the parasite differentiates into a replicative procyclic form to enable survival in its new environment. The lifecycle is completed by migration to the salivary glands and transformation to an adherent epimastigote form, followed by transformation to a detached metacyclic form, which is then competent for transmission into the bloodstream of the mammalian host when the tsetse takes a blood-meal.

Both the procyclic form and bloodstream form of the parasite may be cultured *in vitro*. Reverse genetic approaches have been made possible by constructing cell lines containing T7 and tetracycline-responsive procyclin promoters to drive expression of the selectable marker and test gene respectively [3]. Through adaptation to continuous culture the bloodstream form parasite has become monomorphic, having lost the ability to spontaneously transform to stumpy morphology, but is still considered a relevant model system.

Trypanosomes are one of the most evolutionarily divergent eukaryotes for which there are molecular data [4]. The regulation of gene expression in trypanosomes is distinct from that in most eukaryotes, as, except for key surface molecules in *T. brucei* [5], it does not occur at the transcriptional level. Instead, genes are transcribed in large polycistronic units, with post-transcriptional regulation of mRNA processing and stability used to control mRNA abundance [6]. In *T. brucei* the mRNAs from neighbouring genes will often display distinct developmentally regulated profiles [7], [8]. Additional processes such as regulated protein synthesis, modification and turnover will also contribute to regulated gene expression [9].

The variation in mRNA abundance in *T. brucei* between lifecycle stages and during the differentiation process has recently been examined by three global transcriptomic studies using microarrays [10], [11], [12]. Each found extensive regulation of mRNA abundance occurs between lifecycle stages and at different stages during the differentiation process. To date, there have been no genome-wide comparative proteomic studies between the lifecycle stages in *T. brucei*, and the correlation between mRNA and protein abundance is unclear. We have optimized a procedure for growing *T. brucei* procyclic form cells in conditions suitable for stable isotope labeling by amino acids in culture (SILAC) [13], and here we report a genome-wide comparative proteomic analysis of cultured procyclic form and bloodstream form *T. brucei* cells.

## Results and Discussion

### Applying SILAC to *T. brucei*

**Figure 1:**
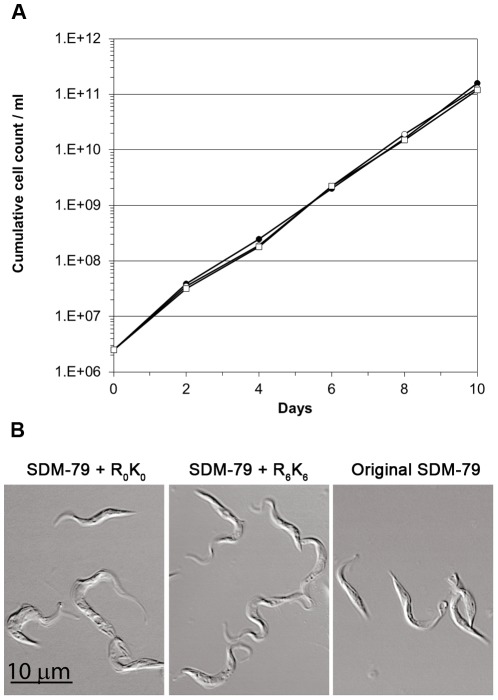
Growth of *T. brucei* procyclic form cells in original SDM-79 and SILAC labelling media. **A.** Cumulative growth curve. Growth in original SDM-79 containing non-dialysed FBS (open squares) is shown in parallel to SDM-79+R_0_K_0_ (open circles) and SDM-79+R_6_K_6_ (closed circles), both containing dialysed FBS. **B.** DIC light microscopy. *T. brucei* procyclic cells grown in original SDM-79, SDM-79+R_0_K_0_ or SDM-79+R_6_K_6_ for ten days were fixed in 4% paraformaldehyde and DIC images acquired on a Zeiss confocal microscope.

The procyclic form *T. brucei* cells were grown in a modified SDM-79 media where L-arginine and L-lysine could be replaced by stable heavy isotopes forms as required for SILAC. Growth curves of procyclic form *T. brucei* cells grown in original SDM-79 [14], modified SDM-79 with normal isotopic abundance L-arginine and L-lysine (SDM-79+R_0_K_0_), or in modified SDM-79 with L-arginine U-^13^C_6_ and L-lysine U-^13^C_6_ (SDM-79+R_6_K_6_) were determined and demonstrated that the division time was unaffected (Fig. 1A). Furthermore, the gross morphology of the cells was unaffected after ten days culture, as judged by DIC light microscopy (Fig. 1B).

If heavy isotope incorporation occurs only by dilution (neglecting protein turnover), then 6–7 cell divisions should produce 96.7–98.3% incorporation. To experimentally assess the efficiency of isotope incorporation, procyclic cells were grown in SDM−79+R_6_K_6_ for 6–7 cell divisions and subjected to analysis by LC-MS/MS. The extent to heavy isotope incorporation was estimated to be 98.8±1.5% by comparing the relative abundance of the major isotopic peak of the heavy (arginine-^13^C_6_/lysine-^13^C_6_) and light (arginine-^12^C_6_/lysine-^12^C_6_) forms of twenty peptides chosen at random. No significant incorporation of proline-^13^C_5_ (by conversion of arginine-^13^C_6_) was observed, most likely because the procyclic growth media is rich in unlabeled proline that would significantly dilute any proline-^13^C_5_ made from arginine-^13^C_6_.

**Figure 2:**
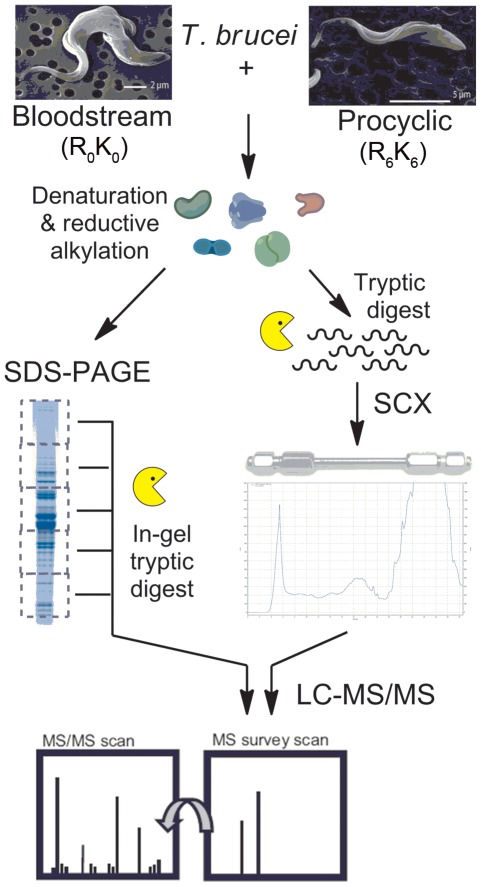
Proteomics workflow. Procyclic cells were cultured in SDM-79+R_6_K_6_ then mixed 1∶1 with either unlabeled procyclic or bloodstream form cells. Sample complexity was reduced prior to LC-MS/MS analysis by either fractionation at the protein level by SDS-PAGE or at the peptide level by SCX chromatography.

To assess the distribution of isotope incorporation across the proteome we mixed an equal number of procyclic cells grown in the presence of normal L-arginine and L-lysine (R_0_K_0_) with cells grown in the presence of L-arginine and L-lysine uniformly incorporating ^13^C (R_6_K_6_) for 6–7 cell divisions and conducted a global proteomic analysis. To ensure maximum coverage of membrane and structural proteins, total protein extracts were prepared using the filter-aided sample preparation technique, which uses complete solubilization with 4% SDS [15]. After denaturation and reductive alkylation the proteins were either fractionated by SDS-PAGE and subjected to in-gel tryptic digest, or digested with trypsin in solution and peptides separated by SCX chromatography (Fig. 2). The use of two orthogonal techniques to fractionate the sample at the protein or peptide level was designed to improve coverage of the proteome.

**Figure 3:**
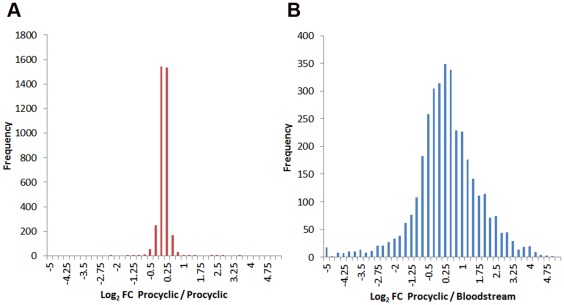
Histograms of Log_2_ fold change. **A.** Procyclic form labeled with heavy isotopes (R_6_K_6_) mixed 1∶1 with unlabeled procyclic form (R_0_K_0_). **B.** Procyclic form labeled with heavy isotopes (R_6_K_6_) mixed 1∶1 with unlabeled bloodstream form (R_0_K_0_).

The eight fractions obtained from SDS-PAGE and ten SCX fractions were subjected to LC-MS/MS in technical duplicates, and the 36 data files analyzed using MaxQuant [16], [17] to search a *T. brucei* 927 protein sequence database. Altogether 248,648 MS/MS spectra were identified, corresponding to 37,051 non-redundant peptide sequences and 4005 protein groups with a false discovery rate of 0.01. The high number of proteins identified (49% of predicted ORFs) validates the sample processing technique. Heavy to light ratios (fold-change, FC) could be determined for a total of 3662 protein groups, with the observed ratios normally distributed about 1 (Log_2_ FC = 0) as expected for a 1∶1 mixture (Fig. 3 A), confirming that efficient labelling had occurred. Comparison of the orthogonal separation techniques revealed that analysis of the SDS-PAGE samples alone was able to quantify ratios for 1639 protein groups, including 114 protein groups not quantified by SCX separation. The SCX samples were able to quantify ratios for 3548 protein groups, including 2023 protein groups not quantified by SDS-PAGE separation. The separation techniques did not show any significant bias towards number of transmembrane domains or the proteins isoelectric point. The SDS-PAGE analysis detected slightly fewer proteins with molecular weight >200 kDa (1.9%) than SCX (2.9%). The higher number of observation made by SCX separation may reflect that, due its higher capacity, approximately ten times as much material was loaded on the SCX column as was possible to resolve by SDS-PAGE. Despite this, SDS-PAGE was still able to quantify as significant number of unique protein groups.

To demonstrate the utility of SILAC to inform biology we conducted a global comparative proteomic analysis of procyclic form and monomorphic bloodstream form *T. brucei*. Cultured bloodstream form cells were grown in the presence of normal L-arginine and L-lysine (R_0_K_0_) and mixed 1∶1 with procyclic form cells grown in the presence of L-arginine and L-lysine uniformly incorporating ^13^C (R_6_K_6_) for 6–7 cell divisions. The cells were detergent solubilized, fractionated by SDS-PAGE and SCX, and analyzed by LC-MS/MS as described above. Altogether 241,537 MS/MS spectra were identified, corresponding to 38,084 non-redundant peptide sequences and 3959 protein groups with a false discovery rate of 0.01. Heavy to light ratios (fold-change) could be determined for a total of 3553 protein groups (Table S1). Comparison of the orthogonal separation techniques revealed that the SDS-PAGE samples alone quantified ratios for 2381 protein groups (272 unique), whilst SCX samples quantified ratios for 3281 protein groups (1172 unique). The observed heavy to light ratios were widely distributed (Fig. 2 B), with 10.6% differentially regulated by more the 5-fold (Log_2_ FC>2.35) between lifecycle stages. These results are analyzed in more detail below.

### Agreement with known biology

**Figure 4:**
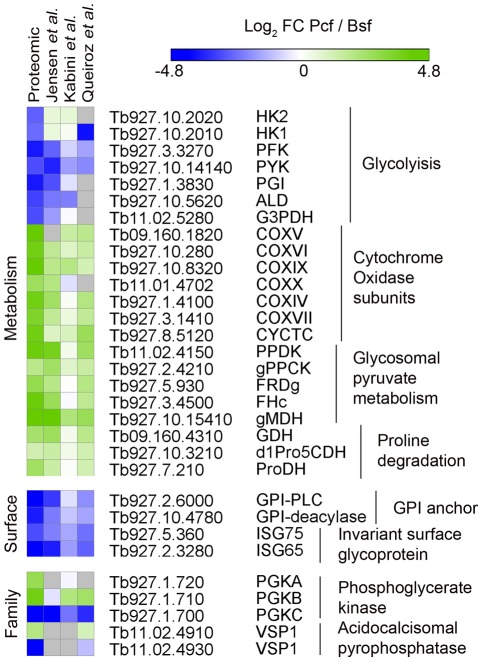
Agreement of comparative proteomic data with known biology. Heatmap showing the Log_2_ FC (procyclic to bloodstream) derived from comparative proteomic data (this study) and previous transcriptomic studies [10], [11], [12]. Grey – not observed. Heatmap generated with GENEE (http://www.broadinstitute.org/cancer/software/GENE-E/).

We initially sought to validate our comparative proteomic data by examining the fold-changes for proteins known to show differential regulation between lifecycle stages (Fig. 4). There are major changes to the energy metabolism between procyclic and bloodstream form cells that occur in response to their differing host environments. Bloodstream form trypanosomes derive their energy from the metabolism of glucose mainly into pyruvate in a glycolytic pathway compartmentalized into a specialized peroxisome called the glycosome [18]. In contrast, procyclic form cells have several alternative pathways for energy. In culture, proline is the major energy source, and although they still metabolize glucose it is mainly into phosphoenol pyruvate, which can be converted by several routes including into acetate in the mitochondrion [19]. In agreement with these observations, the comparative proteomic data shows seven glycolytic enzymes are down-regulated in procyclic form (Log_2_ FC −2.3 to −3.6), whilst seven nuclear encoded subunits of cytochrome oxidase are up-regulated (Log_2_ FC 2.6 to 4.6) [20]. Additional metabolic enzymes that are up-regulated in procyclic form include five enzymes involved in glycosomal pyruvate metabolism (Log_2_ FC 1.8 to 4.1) and three enzymes involved in proline degradation (Log_2_ FC 1.8 to 2.5).

There are major changes to the trypanosome surface coat between lifecycle stages. Bloodstream form cells have a dense surface coat of 5×10^6^ copies of a single GPI anchored Variant Surface Glycoprotein (VSG) dimers and collectively 1×10^5^ copies of Invariant Surface Glycoprotein 65 & 75 (ISG65 & ISG75) family members [21]. Procyclic cells have a surface coat of GPEET and EP procyclins, anchored by GPI structure distinct from that found in the bloodstream form by virtue of inositol acylation which renders it resistant to GPI-PLC.

The VSG variant expressed in our cultured cell line (VSG221, MITat 1.2) is not present in the *T. brucei* 927 genomic database, and, therefore, was not observed in the standard proteomic analysis, nor were we able to observe procyclins due to their resistance to tryptic digestion [22]. In order to observe VSG, the VSG221 sequence (GI:139611) was appended to the protein sequence database. Re-analysis of the data identified VSG221 with 55 unique peptides (82.6% sequence coverage), and confirmed that VSG221 is strongly down-regulated in procyclic form (Log_2_ FC −4.9). We were also able to see that ISG65 and ISG75 (Log_2_ FC −4.3 and −2.8) were down-regulated in procyclic form, as were the enzymes GPI-PLC [23] and GPI deacylase [24] (Log_2_ FC −3.6 and −4.1) involved in stage-specific GPI processing

The ability of the proteomic data to distinguish proteins with high sequence homology was confirmed by its ability to discriminate between two families of enzymes known to be developmentally regulated. It has previously been observed that the two tandemly linked copies of the acidocalcisomal pyrophosphatase *VSP1* (Tb11.02.4910 and Tb11.02.4930) are reciprocally regulated at the mRNA level between bloodstream and procyclic stages [7], [10]. Despite high sequence homology between the two genes the comparative proteomic data were able to discriminate between them based on 4 unique peptides observable for Tb11.02.4910 (Log_2_ FC 2.0) and 3 unique peptides for Tb11.02.4930 (Log_2_ FC −4.1), confirming the transcriptomic observation. The regulation of phosphoglycerate kinase *PGK* mRNA levels are known to vary with the isoform, with *PGKC* (Tb927.1.700) being down-regulated in procyclic form, *PGKB* (Tb927.1.710) being up-regulated in procyclic form and *PGKA* being constitutively expressed in both bloodstream and procyclic form parasites [8], [25]. The comparative proteomic data was able to distinguish the three isoforms with Log_2_ FC −4.5 for PGKC (12 unique peptides) and Log_2_ FC 3.8 for PGKB (8 unique peptides), in agreement with the trend in the observed mRNA change. However, the proteomics data revealed that PGKA was strongly up-regulated in procyclic form (Log_2_ FC 2.8, 12 unique peptides) at the protein level, in contrast with the reported constitutive expression. This observation raises the possibility that the regulation of PGKA abundance may occur independently of mRNA level, adding further complexity to the regulation of PGK isoforms.

### Correlation between proteomic and transcriptomic data

**Figure 5:**
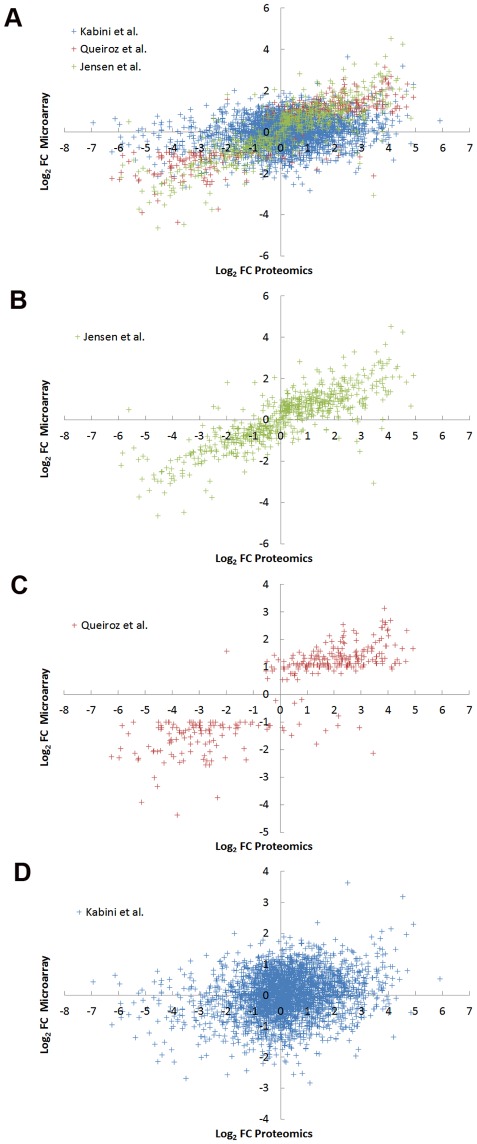
Comparison of Proteomic and transcriptomic data. Scatterplot of the Log_2_ FC (procyclic to bloodstream) derived from comparative proteomic data (this study) and previous transcriptomic studies [10], [11], [12].

The correlation between the comparative proteomic data and the three recently reported *T. brucei* transcriptomic studies was examined (Fig. 5 A–D, and Table S2) [10], [11], [12]. Some of the variation in correlation observed (Fig. 5 A) may be explained by considering the differences between the three studies. A good correlation (0.86) between protein and mRNA abundance was found when comparing to the Log_2_ FC in mRNA between cultured procyclics and cultured bloodstream form cells (pleomorphic ‘genome’ strain TREU927/4) reported by Jensen *et al.* [10] (Fig. 5B). A slightly lower level of correlation (0.83) between protein and mRNA abundance was found when comparing to the Log_2_ FC in mRNA between cultured procyclics and cultured bloodstream form cells (pleomorphic strain AnTat1.1) reported by Queiroz *et al.* [12]. The final study by Kabini *et al.* [11] used animal derived bloodstream form cells (pleomorphic strain AnTat1.1) to examine differentiation to procyclic form up to 48 h after initiation of differentiation, and we have used the Log_2_ FC between 48 h and slender bloodstream form cells. The poor correlation (0.2) with the proteomic data may be reflective of the variability of animal infections and differences between established cultured procyclic form cells and cells 48 h after initiation of differentiation.

Overall, the good level of correlation between the protein and mRNA abundance lends support to the hypothesis that the post-transcriptional regulation of mRNA level is a significant component in the regulation of gene expression in *Trypanosoma brucei*. The fold-changes observed at the protein level are consistently larger (by ∼2-fold) than those observed at the mRNA level, suggesting that either an amplification effect occurs, or the introduction of experimental bias due to differences in effective dynamic range. The level of correlation between the transcriptomic studies of Jensen *et al.* and Queiroz *et al.* of 0.91 is only a slight improvement to their correlation to the proteomic data (0.86 and 0.83 respectively). Of a total of 43 protein ratios showing negative correlation with the mRNA ratio of Jensen *et al.* with a Log_2_ FC>|0.5|, only 8 showed negative correlation with both Jensen *et al.* and Queiroz *et al.* (Table 1). Six of these proteins could be identified with 2 or more unique peptides. Any biological significance of the negative correlation is unclear.

### GO term enrichment

**Figure 6:**
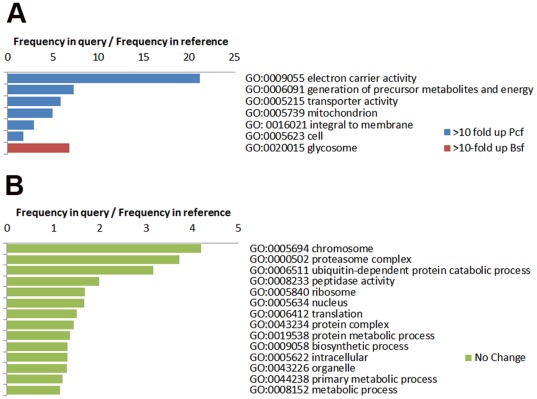
GO term enrichment. **A.** Proteins with greater than ten-fold up- or down regulation, with enrichment P<0.01. **B.** Constitutively expressed proteins, with enrichment P<0.01.

A Gene Ontology analysis was performed using a GO slim set to identify functional classes of genes amongst those that were either more than ten-fold up- or down-regulated (Log_2_ FC≥3.32 or ≤−3.32) at the protein level between the lifecycle stages. Due to the high occurrence of proteins annotated as hypothetical conserved in the *T. brucei* genome only 55 out of the 143 proteins showing either more than ten-fold up- or down-regulation could be analyzed. This may contribute to the fact that the enriched GO terms (P<0.01) are dominated by known biology, *i.e.* changes in metabolism and energy (Fig. 5A). An equivalent analysis was performed using proteins that were constitutively expressed (Log_2_ FC±0.25). Of the 663 constitutively expressed proteins 222 were analyzed, with enriched GO terms (P<0.01) including many core cellular processes, as expected (Fig. 6B).

### Conclusion

We have established SILAC steady-state labeling in procyclic form *T. brucei*, and demonstrated the power of the technique by conducting a global comparative proteomic analysis of cultured procyclic and bloodstream form parasites. This work should be a useful resource for the community as it provides experimental evidence of the expression of a large number of hypothetical conserved proteins and their developmental regulation. The establishment of SILAC in *T. brucei* will enable this powerful technique to be used to refine many further studies such as subcellular fractionations, protein-protein interactions and signaling pathway analysis [26]. To make our data accessible to the scientific community, we have uploaded our study to TriTrypDB, and deposited the LC-MS/MS files into the Proteome Commons Tranche depository, enabling researchers to interrogate the information presented here.

## Materials and Methods

### SILAC SDM-79 and original SDM-79 media preparation

The original SDM-79 medium was obtained from Invitrogen in a powder format *via* the Trypanosome Consortium, kindly organized by Helen Banks in Prof. Keith Gull's lab, Oxford, UK, and prepared according to the original formulation [14]. The powder was hydrated in 5 L Milli-Q water, supplemented with 7.5 mg/L of haemin (from a 10 mg/ml stock in 0.1 M NaOH) and 2 g/L sodium bicarbonate. The pH was adjusted to 7.3 with NaOH and sterile filtered using Stericups 500 (Millipore). Under sterile conditions, heat inactivated and non-dialyzed fetal bovine serum (PAA) was added to final 15% (V/V), glutamax I (Invitrogen) to 2 mM final, and penicillin-streptomycin solution (Invitrogen) at 1∶1,000 dilution. The antibiotics G418 and hygromycin were added at 15 µg/ml and 50 µg/ml, respectively.

SILAC SDM-79 medium (SDM-79−RK) was prepared by Caisson labs according to the original SDM-79 formulation, but depleted in L-Arginine, L-Lysine, L-Glutamine and sodium bicarbonate to allow fresh additions. Prior to use, this media was supplemented with haemin (7.5 mg/L) and sodium bicarbonate (2 g/L), pH adjusted to 7.3 with NaOH and sterile filtered using Stericups 500 (Millipore). Under sterile conditions, heat inactivated and dialyzed fetal bovine serum (10 kDa molecular weight cut-off, PAA) was added to final 15% (V/V), glutamax I (Invitrogen) to 2 mM final, and penicillin-streptomycin solution (Invitrogen) at 1∶1,000 dilution. The SDM-79−RK was supplemented with either normal isotopic abundance L-Arginine and L-Lysine (SDM-79+R_0_K_0_), or with L-Arginine.HCl U-^13^C_6_ and L-Lysine.2HCl U-^13^C_6_ (SDM-79+R_6_K_6_, Cambridge Isotope Labs, UK) at the same concentration as described in the original SDM-79 formulation [14]. The antibiotics G418 and hygromycin were added at 15 µg/ml and 50 µg/ml, respectively.

### Cell culture

Procyclic form *Trypanosoma brucei* clone 29.13.6 cells (kindly provided by Prof. George Cross) were grown at 28°C without CO_2_ in with fully capped culture flasks (BD, non-treated plastic) in original SDM-79.

For the growth curves the *T. brucei* procyclic form cells were washed 3 times in 10 ml SDM-79−RK, and resuspended at 2.5×10^6^ cells/mL in either original SDM-79, SDM-79+R_0_K_0_ or SDM-79+R_6_K_6_. Every 2 days the cells were counted using a Neubauer chamber and phase contrast microscope, and the cultures were diluted 7.7 times. After 10 days samples were collected for analysis by light microscopy.

For SILAC labeling, *T. brucei* procyclic form cells, in log phase of growth were washed 3 times with 10 ml of SDM-79−RK, and resuspended at 2.5×10^6^ cells/mL in either SDM-79+R_0_K_0_ or SDM-79+R_6_K_6_. Cells were passaged every 2 days by diluting about 7.7 fold to obtain ∼2.5×10^6^ cells/mL to enlarge the culture and to reach 6–7 cell divisions under labeling conditions.

Culture adapted strain 427 monomorphic bloodstream form *T. brucei* (variant 221, MITat 1.2) genetically modified to express T7 polymerase and the tetracycline repressor protein, as described by Wirtz *et al.* [3], were cultured in HMI-9T medium [27] containing 2.5 µg/mL G418 at 37°C in a 5% CO_2_ incubator. HMI-9T is a modification of the original HMI-9 that uses 56 µM 1-thioglμycerol in place of 200 µM 2-mercaptoethanol, and contains 10% heat inactivated fetal Bovine serum (PAA).

**Table 1:** Negative correlation between protein and Mrna.

GeneDB ID	Description	Peptides^a^	Proteomics	Jensen *et al.*	Queiroz *et al.*	Kabini *et al.*
Tb09.211.1030	inositol phosphorylceramide synthase	1	−1.99	1.81	1.58	1.35
Tb11.01.8225	hypothetical protein, conserved	4	−0.47	0.92	1.20	0.09
Tb11.02.3210	triosephosphate isomerase	15	−0.31	1.09	1.00	0.07
Tb927.8.1790	hypothetical protein, conserved	3	−0.21	2.06	1.43	1.08
Tb927.7.4110	kinesin, putative	2	0.14	−0.79	−1.32	−0.85
Tb927.8.870	serine/threonine kinase	3	2.16	−0.89	−0.78	−0.78
Tb11.01.8270	zinc finger protein family	19	2.94	−1.54	−1.20	0.00
Tb927.10.8450	glucose transporter 1E	1	3.44	−3.08	−2.14	−0.45

a – number of unique peptides mapped to each protein. Log_2_ FC (procyclic to bloodstream) derived from comparative proteomic data (this study) and previous transcriptomic studies [10], [11], [12].

Cells were harvested by centrifugation and hypotonically lysed at 5×10^9^ cells/mL for 5 min on ice in the presence 0.1 µM 1-chloro-3-tosylamido-7-amino-2-heptone (TLCK), 1 mM benzamidine, 1 mM phenyl-methyl sulfonyl fluoride (PMSF), 1 µg/mL leupeptin, 1 µg/mL aprotinin and Phosphatase Inhibitor Mixture II (Calbiochem). The protein concentration was determined by BCA assay (Pierce) to be ∼5 mg/mL from each cell type. Samples were aliquoted, snap frozen, and stored at −80°C prior to subsequent processing.

### Microscopy

Procyclic form cells at late log phase grown in either original SDM-79, SDM-79+R_6_K_6_ or R0K0 for 10 days were washed in 10 ml phosphate buffer saline at 600×g at 4°C, fixed in 4% paraformaldehyde in phosphate buffered saline at 4°C for 30 min, and placed on a cover slip. After air-drying the cover slips were washed in phosphate buffered saline and mounted onto slides. The differential interference contrast (DIC) images were collected in a Zeiss LSM 700 META confocal microscope.

### Estimating efficiency of SILAC labeling

Procyclic cells grown in SDM−79+R_6_K_6_ for 6–7 cell divisions and hypotonically lysed as described above. To reduce the sample complexity the proteins were fractionated by SDS-PAGE, and a band corresponding to 25–50 kDa molecular weight range was excised and subjected to in-gel tryptic digest prior to analysis by LC-MS/MS. Twenty peptides were chosen at random and the relative abundance of the major isotopic peak of heavy (arginine-^13^C_6_/lysine-^13^C_6_) and light (arginine-^12^C_6_/lysine-^12^C_6_) forms were measured in extracted ion chromatograms using the Excalibur software (Thermo Scientific).

### Filter aided sample preparation

Samples for analysis by mass spectrometry were prepared by modification of the filter-aided sample preparation procedure [15] and fractionated by either SDS-PAGE or strong cation exchange (SCX) chromatography. Samples containing 7.5×10^7^ lysed cells (15 µL) were defrosted and combined according to the experiment design. The combined sample was solubilized by addition of 30 µl buffer A (8% SDS, 200 mM DTT, 200 mM Tris-HCl pH 8.0) followed by vigorous vortexing for 3 min, sonication for 3 min, heating to 95°C for 3 min and a vortexing for a further 3 min. Samples were centrifuged at 16,000×g for 5 min to remove insoluble material, although none was visible.

The solubilized sample was reductively alkylated using the standard FASP I procedure in a 10,000 MWCO horizontal spin filtration unit (Vivascience), and washed into 40 µL of 50 mM ammonium bicarbonate [15]. At this point 10% of the sample was withdrawn for separation by SDS-PAGE prior to in-gel tryptic digest (see below). The remaining sample was digested with 1.5 µg ratio of trypsin gold (Promega) in the filtration unit for 18 h at 37°C. Tryptic peptides were eluted by centrifugation at 16,000×g for 10 min, and the filtration washed with sequentially with 40 µL of 50 mM NH_4_HCO_3_ and 40 µL of 0.5 M NaCl. The combined eluent was desalted using a 50 mg C_18_ cartridge (SepPak, Waters) and lyophilized.

### Strong cation exchange chromatography

Strong cation exchange was performed on an Agilent 1120 compact LC using a 3.0×200 mm 5 µm polysulfoethyl aspartamide column (Poly LC) with a flow rate of 350 µL/min and detection at 220 nm. Dried peptides were dissolved in 200 µL of solvent A (10 mM KHPO_4_ pH 3.0, 30% MeCN) and separated by salt gradient consisting of 5 min at 100% solvent A, a 22.5 min gradient to 42% solvent B (solvent A+0.6 M KCl, 7.5 min gradient to 100% B, 5 min at 100% B, and a 5 min gradient to 100% A. Fractions of 700 µL were collected throughout the run and combined into 10 fractions of equal peptide content based on their absorbance at 220 nm. Combined fractions were desalted using microcolumns containing 1 mg Oligo R3 (AppliedBiosystems) in C_18_ ZipTips (Millipore) and lyophilized prior to analysis.

### Polyacrylamide gel electrophoresis

For SDS-PAGE, ∼5 µg of reductively alkylated sample was subjected to electrophoresis on a NuPAGE bis-Tris 4–12% gradient acrylamide gel under reducing conditions and stained with Simply Blue colloidal Coomassie (Invitrogen). The sample lane was divided into eight bands that were excised, and subjected to in-gel digestion for 18 h at 37°C with 12.5 µg/mL trypsin gold (Promega) in 10 mM NH_4_HCO_3_, 10% MeCN. Tryptic peptides were recovered in 45% MeCN, 1% formic acid and lyophilized prior to analysis.

### Mass spectrometry data acquisition and processing

Liquid chromatography tandem mass spectrometry was performed by the Proteomic Facility at the University of Dundee. Liquid chromatography was performed on a fully automated Ultimate U3000 Nano LC System (Dionex) fitted with a 1×5 mm PepMap C_18_ trap column and a 75 µm×15 cm reverse phase PepMap C_18_ nanocolumn (LC Packings, Dionex). Samples were loaded in 0.1% formic acid (buffer A) and separated using a binary gradient consisting of buffer A and buffer B (90% MeCN, 0.08% formic acid). Peptides were eluted with a linear gradient from 5 to 40% buffer B over 65 min. The HPLC system was coupled to an LTQ Orbitrap Velos mass spectrometer (Thermo Scientific) equipped with a Proxeon nanospray ion source. The mass spectrometer was operated in data dependent mode to perform a survey scan over a range 335–1800 m/z in the Orbitrap analyzer (*R* = 60,000), with each MS scan triggering ten MS^2^ acquisitions of the ten most intense ions. The Orbitrap mass analyzer was internally calibrated on the fly using the lock mass of polydimethylcyclosiloxane at *m*/*z* 445.120025.

Data was processed using MaxQuant [16] version 1.2.2.5 which incorporates the Andromeda search engine [17]. Proteins were identified by searching a protein sequence database containing *T. brucei brucei* 927 annotated proteins (Version 3.2, downloaded from TriTrypDB [28], http://www.tritrypdb.org/) supplemented with frequently observed contaminants (porcine trypsin, bovine serum albumins and human keratins). Search parameters specified a MS tolerance of 5 ppm, a MS/MS tolerance at 0.5 Da and full trypsin specificity, allowing for up to three missed cleavages. Carbamidomethylation of cysteine was set as a fixed modification and oxidation of methionines, N-terminal protein acetylation and N-pyroglutamate were allowed as variable modifications. Peptides were required to be at least 6 amino acids in length, and false discovery rates (FDRs) of 0.01 were calculated at the levels of peptides, proteins and modification sites based on the number of hits against the reversed sequence database. SILAC ratios were calculated using only peptides that could be uniquely mapped to a given protein.

### Bioinformatic analysis

Processed mass spectrometery data was further analyzed using the information contained in TriTrypDB (http://www.tritrypdb.org) [28]. Gene ontology (GO) term enrichment was carried out using GOTools (http://genome.crg.es/GOToolBox/) [29] using a generic GO slim set containing 11 additional terms to capture trypanosome biology [30]. To make our data accessible to the scientific community, we uploaded our study to TriTrypDB (http://www.tritrypdb.org), and deposited the LC-MS/MS files into the Proteome Commons (http://www.proteomecommons.org) Tranche depository (#6nVGofIEQu6D4odoX8aAdpUngsx1fAv43g8UN2w23Bb/dXd6zBwaqq7SQKQcH7Mf05dGtfye0vl8pnrH3muce8eA67EAAAAAAAAcbw =  = ), enabling researchers to access the data presented here.

## Supporting Information

Table S1 Comparative proteomic data.
(XLS)

Table S2 Comparison of proteomic and transcriptomic data.
(XLS)
